# Targeted ^1^H NMR metabolomics and immunological phenotyping of human fresh blood and serum samples discriminate between healthy individuals and inflammatory bowel disease patients treated with anti-TNF

**DOI:** 10.1007/s00109-021-02094-y

**Published:** 2021-05-21

**Authors:** Sara Notararigo, Manuel Martín-Pastor, Juan E. Viñuela-Roldán, Adriano Quiroga, J. Enrique Dominguez-Munoz, Manuel Barreiro-de Acosta

**Affiliations:** 1 Instituto de Investigación Sanitaria de Santiago (IDIS), Complejo Hospitalario Universitario de Santiago (CHUS), Servicio Gallego de Salud (SERGAS), 15706 Santiago de Compostela, A Coruña Spain; 2 Unidade de Resonancia Magnética, Área de Infraestruturas de Investigación, CACTUS, University Santiago de Compostela, 15782 Santiago de Compostela, A Coruña Spain

**Keywords:** NMR metabolomics, IBD stratification, IBD metabolomic profiling

## Abstract

**Abstract:**

Inflammatory bowel disease is a multifactorial etiology, associated with environmental factors that can trigger both debut and relapses. A high level of tumor necrosis factor-α in the gut is the main consequence of immune system imbalance. The aim of treatment is to restore gut homeostasis. In this study, fresh blood and serum samples were used to identify biomarkers and to discriminate between Crohn’s disease and ulcerative colitis patients under remission treated with anti-TNF. Metabolomics based on Nuclear Magnetic Resonance spectroscopy (NMR) was used to detect unique biomarkers for each class of patients. Blood T lymphocyte repertories were characterized, as well as cytokine and transcription factor profiling, to complement the metabolomics data. Higher levels of homoserine-methionine and isobutyrate were identified as biomarkers of Crohn’s disease with ileocolic localization. For ulcerative colitis, lower levels of creatine-creatinine, proline, and tryptophan were found that reflect a deficit in the absorption of essential amino acids in the gut. T lymphocyte phenotyping and its functional profiling revealed that the overall inflammation was lower in Crohn’s disease patients than in those with ulcerative colitis. These results demonstrated that NMR metabolomics could be introduced as a high-throughput evaluation method in routine clinical practice to stratify both types of patients related to their pathology.

**Key messages:**

- NMR metabolomics is a non-invasive tool that could be implemented in the normal clinical practice for IBD to assess beneficial effect of the treatment.
- NMR metabolomics is a useful tool for precision medicine, in order to sew a specific treatment to a specific group of patients.
- Finding predictors of response to IFX would be desirable to select patients affected by IBD.
- Immunological status of inflammations correlates with NMR metabolomics biomarkers.

**Supplementary Information:**

The online version contains supplementary material available at 10.1007/s00109-021-02094-y.

## Introduction

Inflammatory bowel disease (IBD) is a chronic intestinal inflammatory condition, of unidentified etiology, that comprises two major distinct pathologies: Crohn’s disease (CD) and ulcerative colitis (UC); CD can affect both the ileum and colon while UC is limited to the colon. There is also a third group of individuals positive to the IBD test that are categorized as having indeterminate colitis; however, they only represent 5% of total IBD [1].

Genetic and environmental factors, immune system imbalance, and altered microbiota are considered the main causes of this disease. In the healthy condition, the gastrointestinal tract is colonized by a microbiota that plays an important role in gut homeostasis through the interaction with innate immune system cells. Antigen-presenting cells (APC), such as dendritic cells (DC) or intestinal epithelial cells, are able to sense the extra-luminal environment and cooperate with the gut adaptive immune system to maintain a tolerogenic response by activation of the CD4^+^ T regulatory subset, expressing forkhead box P3 (FoxP3) transcription factor, and thereby secreting interleukin-10 into the gut *lamina propria*. In IBD pathogenesis, crosstalk between the gut immune system cells and the microbiota is altered, leading to the disruption of the gut barrier and translocation of microbiota to the *lamina propria* that triggers Th1/Th2 and Treg/Th17 imbalance that culminates in chronic inflammation [2]. Aberrant T cell activation and proliferation are one of the mechanisms responsible for IBD, where high amounts of pro-inflammatory cytokines such as tumor necrosis factor-α (TNF-α), interleukin-17 (IL-17), or interferon-γ (IFN-γ) are released in the gut epithelium by activated Th1, or Th17 [3]. It is not clear if microbiota translocations are the cause or the effect of aberrant inflammation since both the high levels of TNF-α and Th1 over activation determine aberrant proliferation and autoimmunity.

Since TNF-α plays a pivotal role in IBD pathogenesis, several anti-TNF treatments have been introduced into clinical practice to decrease TNF-α concentration in the tissue. They are able to induce and maintain clinical remission, and to decrease surgery and hospitalization [4]. Unfortunately, only 30% of treated patients are considered primary responders, as many of them develop immunogenicity to the treatment with the consequent loss of response [5]. The causes are variable and often one treatment has to be changed for another. In order to explain the causes of this loss of response and the consequent relapses, it is important to obtain a reliable methodology to detect treatment predictors.

In this regard, flow cytometry and NMR metabolomics offer the possibility of developing a high-throughput methodology for a preliminary analysis to improve clinical IBD diagnosis, to select markers to assess the treatment effectiveness and also to minimize invasive tests such as colonoscopy that are normally used in clinical practice.

Flow cytometry is a widely used quantitative technique used in immunology and clinical immunology to monitor inflammation. It can be used to detect CD4^+^ T cell absolutes in HIV patients [6], immunophenotyping in leukemia and lymphoma [7], immunodeficiency diagnosis [6], and solid tumors [7]. Some advantages of flow cytometry over other techniques, such as qPCR, include the ease of sample preparation, cost-effectiveness, high-throughput capability, and no requirement for validation, and finally, it provides a quantitative phenotypic measurement of the targeted proteins. The basis of flow cytometry is the ability of a cocktail of antibodies to bind specifically to their counterpart proteins, thus allowing the identification of selected immune cell types from a complex sample matrix (as would be the case of fresh blood), thus avoiding the need for further isolation steps. Additionally, flow cytometry does not require a large amount of sample, for instance, to analyze the whole leucocyte repertory. Thus, this procedure is a good candidate for routine clinical practice. Moreover, its versatility enables it to detect proteins at cytosolic or nuclear level with the same precision and robustness as surface staining [8].

NMR is an analytical tool widely used in metabolomics for biomedical applications [9, 10]. Despite its relative low sensitivity compared to other techniques such as mass spectrometry, NMR is a non-invasive, non-destructive, highly reproducible, and high-throughput technique that allows the simultaneous quantification of many organic compounds (metabolites) present in biofluids such as urine, feces, or serum/plasma, without requiring prior purification [11]. The combination of NMR metabolomics with sophisticated bioinformatics and mathematical modeling is able to provide “snapshots” of a patient during the course of a disease and its treatment [9]. The identification of disease markers in a biofluid not only offers practical clinical information concerning the risk, diagnosis, and/or prognosis of the disease [11, 12] but also detects altered metabolic pathways that could lead to a better understanding of the disease and/or to select a plausible target for a therapy. Some successful examples in which NMR metabolomics has proven useful in the detection of disease biomarkers include cancer [12, 13] including colorectal cancer, [14], IBD [15–17], CD [18, 19], and UC [19] just to refer to some recent studies. Moreover, it can replace invasive diagnostics such as colonoscopy [20].

In this work, NMR metabolomics and flow cytometry were used to differentiate between CD and UC patients under remission and treated with IFX, by analyzing the results in blood serum and fresh blood samples, respectively. The main goal is to achieve a reliable methodology to stratify patients that could be useful in clinical decision-making based on molecular markers.

## Material and methods

A case-control study was performed. Inclusion criteria were IBD patients in clinical remission under maintenance with infliximab (IFX) treatment. The IBD diagnosis was based in ECCO (European Crohn and colitis criteria) guidelines. After informed consent, blood sample were obtained in CD and UC patients just before IFX infusion and in healthy controls (CTRL). CD patients were divided into subgroups based on the Montreal Classification, according to the affected gut region (ileocolic (IC), ileum, and colon). Clinical and biological data (fecal calprotectine, C-reactive protein (CRP)) was recorded in all patients.

This study passed the ethical approval of CEIC 2018.410. All the measurement methods were carried out according to the approved guidelines. Informed consent was obtained from all participants.

### Cell surface phenotyping

Surface staining (phenotyping) was performed using fresh peripheral blood collected in the lavender tube with EDTA, BD Vacutainer. A 150-μL sample of fresh blood was used for each test tube (Th1, Th17, Treg,). Blood was incubated with a specific antibody mix for each population, for 30 min in the dark.

Th1: FITC anti-human CCR5 (BD), PE anti-human CXCR3 (Biolegend), APC anti-human CD4 (BD);

Th17: FITC anti-human CCR6 (Biolegend), PE anti-human CD161 (BD), APC anti-human CD14 (BD);

Treg: FITC anti-human CD4 (BD Pharmigen), PE anti-human CD25 (BD Pharmigen), APC anti-human CD127 (Biolegend).

Subsequently, FACS lysing (BD) was added to remove red blood cells (15 min, in darkness at room temperature). Finally, cells were washed with cold FACS Flow (BD) then resuspended in 1 mL of FACS Flow (BD). Ten thousand gated CD4+ cells were acquired by Cell-Quest four colors Facs Calibur cytometer (BD). The analysis of the results was carried out using the FlowJo software v.8.4.

### Peripheral blood mononuclear cell isolation

Fresh blood samples were collected in sodium heparin 10-mL tubes (BD Vacutainer*®*) to obtain peripheral blood mononuclear cells (PBMCs). A Ficoll gradient was performed with lymphocyte isolation solution according to the manufacturer’s (Rafer) instructions. Briefly, 3 mL of Ficoll was added to a graduated centrifuge tube; then, the blood sample was mixed in the proportion 1:2 with PBS pH 7.4 (Gibco), and was gently dispensed over the Ficoll to 12 mL final volume. The preparation was centrifugated at room temperature during 20 min at 2300 rpm without subsequent braking in a Heraeus Labfuge 400 centrifuge. The interphase containing PBMCs was gently isolated with a plastic pipette. The cell solution was washed twice with RPMI media to avoid platelet contamination and then centrifuged at 1000 rpm during 7 min at room temperature using the same centrifuge.

### Intracellular staining

PBMCs were seeded into a 10-mL sterile tube, containing 1 mL of RPMI media (Gibco) containing penicillin/streptomycin 10.000 U/10 mg (Sigma-Aldrich), 10% fecal bovine serum (FBS) (Gibco), at a final concentration of 5 × 10^5^ cell mL^−1^. To stain intracellular cytokines, cells were activated with 10 ng/mL phorbol 12-myristate 13-acetate (PMA) (Sigma-Aldrich), and 1.25 pM calcium ionophore (Sigma-Aldrich) during 2 h at 37 °C in a CO_2_ incubator (Heraeus 6000). Then, Brefeldin A was added to the cell solution, to the final concentration of 5 μg·mL^−1^ (Sigma-Aldrich) and incubated overnight at 37 °C, in a CO_2_ incubator [7, 21].

Cells were fixed and permeabilized with Immunostep Fix and Perm Kit, adding solution A (fixative) and incubated for 15 min at room temperature. Then, cells were washed with Facs Flow buffer (BD) and centrifugated at room temperature during 7 min at 1700 rpm in the Heraeus Labfuge 400. Solution B (permeabilization) was added to the cells, with conjugated intracellular antibody cocktail (FITC Mouse Anti Human IFN-γ (BD); PE Mouse Anti Human TNF-α (BD Pharmigen); PerCP-Cy5 Mouse Anti Human CD3 (BD); Anti IL-17A-APC Human (Miltenyi Biotec); at room temperature during 30 min in the dark). Cells were washed with Facs Flow buffer (BD) and resuspended in 1 mL Facs Flow buffer (BD). Cells were screened with Facs Calibur (BD), and analyzed with FlowJo software v8.7.

### Intranuclear staining

To determine PBMC transcription factor activation with flow cytometry, we used Pharmigen™ Transcription factor buffer set (BD) as recommended by the manufacturers. We seeded in a 10-mL sterile tube to a final concentration of 5 × 10^5^ cell mL^−1^ in 1 mL of RPMI media (Gibco) and completed with penicillin/streptomycin 10.000 U/10 mg (Sigma-Aldrich) and 10% fetal bovine serum (FBS) (Gibco). Surface staining was performed with PerCP-Cy5 Mouse Anti Human CD3 (BD) during 30 min at 4 °C. Cells were fixed and permeabilized with 1 mL Fix and Perm solution at 4 °C during 40–50 min protected from light. Cells were washed twice with Perm/wash buffer, then centrifugated at room temperature during 7 min at 1700 rpm in a Heraeus Labfuge 400.

Intranuclear staining was carried out with an antibody cocktail resuspended in Perm/Wash buffer (APC Mouse Anti Human Fox-P3 (Pharmigen) PE Mouse Anti-human T-bet (BD) and Alexa Fluor 488 Mouse Anti Human Ror-γt (BD)) at 2 °C during 40–50 min in the dark. After another washing step, cells were resuspended in 1 mL Facs Flow buffer (BD). Cells were ready to be acquired by flow cytometry with Facs Calibur (BD) and analyzed with FlowJo software v8.7.

### Serum isolation

A fresh blood sample was collected in a SST II Advance 5-mL tube BD Vacutainer®. After 30 min at RT, the sample was centrifugated at 2800 rpm during 10 min at RT in a Heraeus Labfuge 400; then, the resulting serum was aliquoted into microtubes and stored at −80 °C.

### NMR sample preparation

NMR serum samples were prepared according to Standard Operation Procedures (SOPs) [22, 23]; briefly, 300 μL serum and 300 μL of 0.075 M NaH_2_PO_4_ PBS buffer pH 7.4 (Gibco) were mixed in a microtube, then centrifugated at 14,000×*g* for 10 min at 4 °C to remove any precipitate. Four hundred microliters of this solution was mixed with 50 μL of a solution of sodium 3-(trimethylsilyl)-propionic acid-D_4_ (TSP) (Sigma-Aldrich) prepared in D_2_O (TSP final concentration 0.430 mM) and then transferred to a standard 5-mm OD NMR tube for the measurement.

### NMR data collection

NMR experiments were conducted at 25 °C on a *Bruker NEO* 17.6 T spectrometer (proton resonance 750 MHz), equipped with a ^1^H/^13^C/^15^N triple resonance probe with shielded PFG z-gradient. The spectrometer was equipped with a SampleCase*™* sample changer with capacity for 24 samples and refrigeration of the samples at 5 °C until measurement. The NMR spectra of each sample were measured no later than 6 h after sample preparation. The spectrometer control software was TopSpin v4.0. All the spectra were processed with MestreNova v14.0 software (Mestrelab Research Inc.).

NMR spectrum was measured for each serum sample of the groups ILEOCOLIC, ILEUM, COLON, UC, and CTRL. Three types of 1D proton spectra were obtained for each sample: 1D_^1^H_noepresat (^*1*^*H*), 1D ^1^H_cpmgpresat (^*1*^*H_T*_*2*_), and 1D ^1^H_diffusion-filter-presat (^*1*^*H_Dfilter*). They were measured according to Standard Operation Procedures (SOPs) [11, 23, 24]. Each 1D NMR spectrum was processed and referenced to the TSP peak (δ_TSP_^1^Η = 0 ppm).

The 1D NMR spectra of the same type obtained for the serum samples (i.e., ^*1*^*H,* ^*1*^*H_T*_*2*_, or ^*1*^*H_Dfilter*) were analyzed twice, corresponding to the methods of fingerprinting and targeted profiling [23]. In both analyses, the signals in each spectrum are integrated using the data reduction method known as bucketing integration or data binning [11]. The method measures the area under the curve of a spectrum at a series of equidistant portions called buckets of a fixed width of 0.04 ppm. The buckets analyzed in this work covered the complete ^1^H-NMR spectral region from −5 to 14 ppm without overlap (i.e., bucketing integration with equidistant binning). The buckets located near the two edges of the spectrum that are depleted of signals were discarded. Similarly, buckets in the proximity of the water peak at ca. 4.7 ppm can be affected by the water suppression method (presaturation) and were also discarded. The bucketing integration was applied identically for the strategies of fingerprinting and targeted profiling. The only difference is that for targeted profiling a step of peak alignment was applied prior to the bucketing integration which serves to eliminate the small differences of chemical shift occurring for the signal of a given metabolite along the series of spectra of the samples. In both strategies, the absolute integral of each bucket was normalized to achieve virtual constant concentration which permits the direct comparison between samples eliminating their differences in dilution. Such normalization consisted in dividing the absolute integral of each bucket by the sum of the absolute integrals of all the buckets in the same spectrum [11]. The strategy of targeted profiling is intended to be specific to the metabolite signal intensity, which in NMR is proportional to its actual abundance in the sample. The strategy of fingerprinting is sensitive to the signal intensity as well as to other possible effects capable of altering the chemical shifts in a sample (e.g., intermolecular interactions and salinity effects) [11]. MestreNova software was used for processing and analysis of the spectra using the available tools for peak alignment and bucketing integration. Each NMR spectrum of a serum sample provided a vector of 220 bucket integrals for fingerprinting analysis and another 220 bucket integrals for targeted analysis that were analyzed independently.

### Statistical analysis

The statistical calculations were carried out with software MetaboAnalyst v3.0 (www.metaboanalyst.ca) [25]. The comparison of the NMR spectra of a number of samples of two or more of the groups established (i.e., groups ILEOCOLIC, ILEUM, COLON, UC, and CTRL) consisted in the analysis of their respective vectors of bucket integrals by using both univariant and multivariant statistics. For a given type of NMR spectrum and method of bucketing integration (fingerprinting or targeted profiling), a table was built with the 220 buckets of every sample belonging to the selected groups. Prior to the statistical analysis, the table containing the vectors of bucket integrals of the samples selected was submitted to an operation of normalization by division by the total sum of the 220 buckets, and then submitted to auto-scaling (mean centering and variance scaling).

#### Multivariant statistical analysis

Two types of multivariant statistical analysis (MSA) methods were applied to the NMR bucket data. The first method is the unsupervised principal component analysis (PCA) which was used for the detection of anomalous or outlier samples that have to be discarded because they do not satisfy the criterium of quality, possibly due to errors during sample collection, preparation, storage, and/or NMR measurement. The NMR data of the samples that were not discarded by PCA were subsequently analyzed by the MSA method of Orthogonal-Partial Least Squares-Discriminant Analysis (OPLS-DA). It is a supervised method of classification; the information of the group of each sample was introduced to build a statistical model of sample classification by groups. Based on their pattern of intensities, those spectral buckets that best discriminate between groups provided the highest values of VIP scores and loading factors in OPLS-DA. A random permutation test with 200 permutations was performed with the derived OPLS-DA model to confirm the robustness of the method, resulting in the reported values of *R*^2^ and *Q*^2^. Cross-validation was applied to the OPLS-DA model obtained to determine its accuracy and to derive the confusion matrix of the classification achieved.

Univariant analysis was carried out to study the signal intensity of selected NMR buckets with the highest VIP scores and loading factors found in the MSA calculation by OPLS-DA. They are the buckets with the maximum relevance for the group classification achieved by the MSA method considered. The selected buckets were those with a loading factor comprised in a range from the maximum down to 50% of this value. The distribution of NMR intensities of each one of the selected buckets was represented as Box-plots and the *p* value of the distribution was calculated with the unpaired *t* test. The normality of the data was tested by the Kolmogorov-Smirnov normality test. If data underwent Gaussian distribution performed with Welch’s correction, assuming standard deviation was not equal, or otherwise, the Mann-Whitney test was used.

Metabolite assignment was carried out for each NMR bucket that satisfied the same criterion of selection of the univariant analysis, to identify plausible candidates for biomarkers of the disease. A representative spectrum of the classification was loaded in ChenoMX NMR Suite software v8.5 (Chenomx, Inc.). The experimental signal profile and intensity at the chemical shift of the bucket were then matched to the ^1^H patterns of the 750 MHz metabolite database included in this software. A metabolite candidate is obtained when the bucket chemical shift, intensity, and signal profile (i.e., pattern of J couplings) can be matched to a given signal of a metabolite in the spectral database, and simultaneously, other additional ^1^H peaks of the metabolite match or are compatible with the experimental spectrum. Only those metabolites with favorable matching and that could be assigned unambiguously are reported. The assignment of the metabolites found was finally tested for consistency with the 2D CLIP-COSY-presat [26] and 2D TOCSY-presat [11] spectra that were measured for one of the samples in any of the groups under study.

## Results

### Participants

Patients with IBD in clinical remission under anti-TNF treatment (patients, *n* = 27) were consecutively included. Patients were divided into two groups according to the Montreal classification:

- Crohn disease (CD, *n* = 18, divided into 3 subgroups depending on the disease localization in ILEUM, *n* = 8; COLON, *n* = 4; and ILEOCOLIC, *n* = 6) and
- Ulcerative colitis (UC, *n* = 9) with average age of 45 years from 19 to 69, and equal sex representation.
- The healthy control group (CTRL, *n* = 10) were healthy individuals with no IBD or irritable bowel syndrome.

Regarding CD patients, 50% of patients presented inflammatory symptoms (B1), 11% structuring (B2), and 39% penetrating (B3). Regarding the UC location, 11 were E1, 22% E2, and 67% E3. Thirty-three percent of CD patients had suffered previous surgery, but none of UC patients.

The classification of the patients is given in Table 1. All the patients were under treatment with anti-TNF with infliximab (IFX), for a mean time of 5.9 ± 3.7 years. At the time of inclusion, the mean of fecal calprotectin was 234.5 μg/g and the mean CRP was 0.34 mg/dL. Some patients were also under one additional treatment with mesalazine, a colon cancer protector, or with azathioprine, salazopyrine, or methotrexate to protect against immunogenicity. These extra treatments in principle do not affect the efficacy of IFX.

**Table 1:** Clinical classification and demographic information of the patients included in the pilot study

Classification	Segment affected	Sex	Age	Treatment
Crohn disease	Ileum	M	29	IFX
Crohn disease	Ileum	M	34	IFX + AZA
Crohn disease	Ileum	M	26	IFX
Crohn disease	Ileum	M	50	IFX
Crohn disease	Ileum	M	42	IFX
Crohn disease	Ileum	M	62	IFX
Crohn disease	Ileum	F	54	IFX + AZA
Crohn disease	Ileum	M	48	IFX
Crohn disease	Colon	F	67	IFZ + salazopyrina
Crohn disease	Colon	M	43	IFX + AZA
Crohn disease	Colon	F	20	IFX
Crohn disease	Colon	M	41	IFX
Crohn disease	Ileocolic	F	19	IFX
Crohn disease	Ileocolic	F	21	IFX + AZA
Crohn disease	Ileocolic	F	65	IFX + MTX
Crohn disease	Ileocolic	F	20	IFX + AZA
Crohn disease	Ileocolic	F	24	IFX
Crohn disease	Ileocolic	M	43	IFX + AZA
Ulcerative colitis	Colon	M	61	IFX
Ulcerative colitis	Colon	F	69	IFX + 5ASA
Ulcerative colitis	Colon	M	65	IFX
Ulcerative colitis	Colon	M	29	IFX + 5ASA
Ulcerative colitis	Colon	F	62	IFX + 5ASA
Ulcerative colitis	Colon	F	72	IFX
Ulcerative colitis	Colon	M	72	IFX + AZA
Ulcerative colitis	Colon	M	34	IFX
Ulcerative colitis	Colon	F	36	IFX

*M* male, *F* female, *IFX* infliximab, *AZA* azathioprine, *5ASA* mesalazine, *MTX* methotrexate

### Peripheral blood T lymphocyte phenotyping in CD and UC patients

**Fig. 1:**
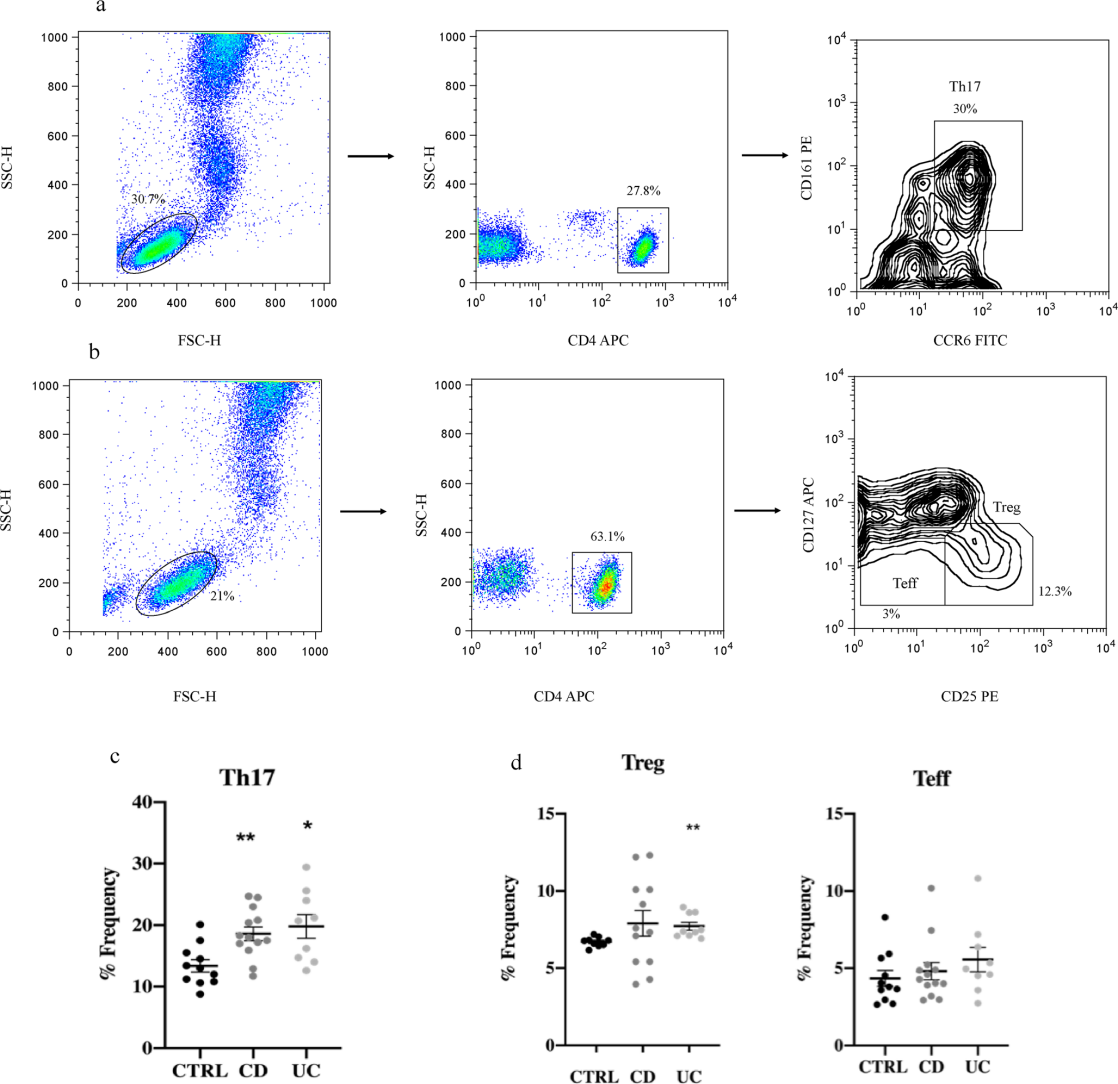
CD4^+^ T lymphocyte phenotyping. **a** Representative cytometric pseudo-color and contour plot from a UC patient. CD4^+^ T cells gated forward side were 27.8%. Total amount of Th17 CD4^+^CCR6^+^CD161^+^ was 30%. **b** Representative cytometric pseudo-color and contour plot from a CD patient. CD4^+^ T cells gated forward side were 63.1%, of which Treg were 12.3%, and Teff 3%. **c** Scatter plot of the Th17 distribution with SEM represented. Data passed the Kolmogorov-Smirnov normality test. Unpaired *t* test with Welch’s correction showed for both groups a significant difference vs. CTRL with a *p* < 0.0021 for CD and *p* < 0.0121 for UC. **d** Scatter plot, mean with SEM represented. The Kolmogorov-Smirnov normality test was applied. Unpaired *t* test with Welch’s correction showed a significant difference only for the UC group with a *p* < 0.0043 vs. control for CD4^+^CD127^+^CD25^+^ Treg

CD4^+^ T cell subsets such as Th1, Th17, and Treg were phenotyped by flow cytometry (Fig. 1 and S1), in order to detect whether or not a subpopulation was involved in the inflammation process. For all the cell subsets, the first gate employed was done by measuring size FSC-H vs. complexity SSC-H parameters, to identify the initial total amount of lymphocytes. To identify CD4^+^ T cells (or CD3^+^ T cells), the cells were gated forward side: SSC-H vs. CD4^+^ or CD3^+^ (Fig. S1a and 1a, 1b).

CD4^+^ T lymphocyte cell surface phenotyping revealed that CD and UC patients had a different distribution pattern of cell populations, as depicted in Fig. 1a, b and S1.

CD4^+^CCR6^+^CD161^+^Th17 subpopulation resulted in a significant difference vs. CTRL with a *p* < 0.0021 for CD and *p* < 0.0121 for UC. This result reveals an ongoing pro-inflammatory activity of immune system cells, especially in UC patients (Fig. 1c).

CD4^+^ Treg cells only showed a significant difference between UC and healthy control groups for the subpopulation CD4^+^CD127^+^CD25^+^ with a *p* < 0.0043 (Fig. 1d). CD patients were distributed in a wider range with a tendency to distribute into two groups. No significant differences were found for effector (Teff) cells (Fig. 1d).

### CD3^+^ T lymphocyte cytokine production

**Fig. 2:**
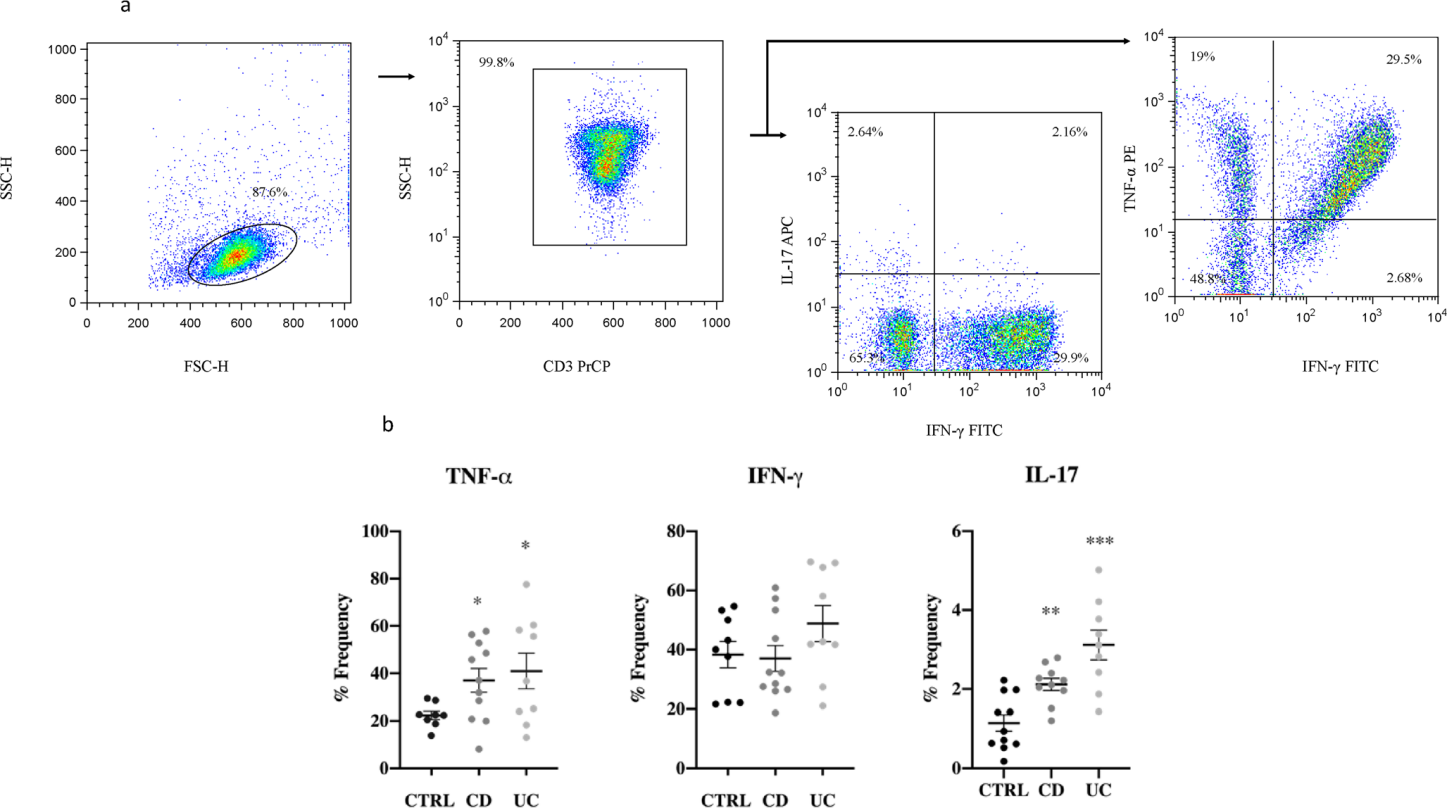
CD3^+^ T cell cytokine production. **a** Representative cytometric pseudo-color plot, from an inflamed UC patient. Gating on CD3^+^ T cell forward side are 99.8%. The percentage of frequency of producing IL-17 was 4.8%, of TNF-α was 48.5%, and of INF-γ was 32.8% respectively. **b** Scatter plot mean with SEM represented. All data underwent the Kolmogorov-Smirnov normality test. For parametric data, the unpaired *t* test with Welch’s correction was applied; for non-parametric data, the Mann-Whitney test was used. TNF-α resulted in a significant difference vs. control in both CD (*p* < 0.01) and UC (*p* < 0.03). No significant difference was observed for INF-γ production. Only IL-17 production showed a significant difference in CD (*p* < 0.0015) with an even higher production of UC (*p* < 0.0006). These results indicate that CD and UC manifest different phenotypes, and that UC patients seem to have a higher ongoing inflammation rate

The ability to produce proinflammatory cytokines was tested in the whole T lymphocyte population by gating in CD3^+^ T cells, thereby including both subtypes: CD4^+^ T helper and the T cytotoxic CD8^+^ (Fig. 2a).

The overall results indicate that CD and UC patients produce a significantly increased amount of TNF-α with a *p* < 0.01 and *p* < 0.03, and also higher IL-17 levels, with a *p* < 0.0015 and *p* < 0.0006, respectively. In line with our previous results, UC demonstrated a higher inflammation rate with respect to CD. No statistical difference was found for IFN-γ production in either group of patients.

### T lymphocyte transcription factor expression

**Fig. 3:**
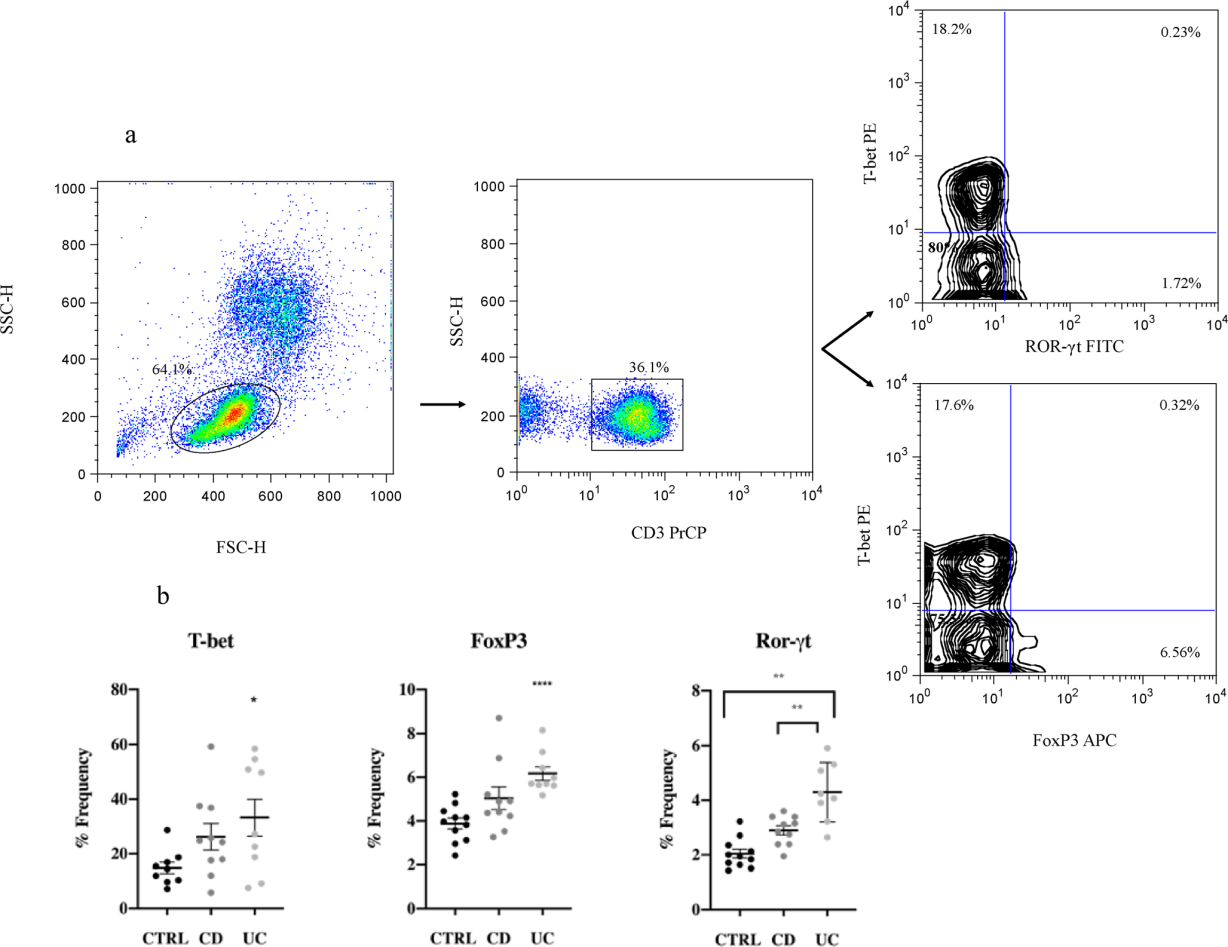
CD3^+^ T cell transcription factor expression. **a** Representative cytometric pseudo-color and contour plot, from an inflamed UC patient. CD3^+^ T cells were gated forward side, with a total percentage of 36.1%. T-bet expression was 18%, FoxP3 was 6.88%, and Ror-γt was 1.95%, with respect to the total amount of CD3^+^ T lymphocytes. **b** Scatter plot mean with SEM represented. All data underwent Kolmogorov-Smirnov normality test. For parametric data, unpaired *t* test with Welch’s correction was applied; for non-parametric data, Mann-Whitney test was applied. T-bet and Fox P3 showed a significant difference only for UC vs. control, with *p* < 0.0272 and *p* < 0.0001, respectively. Ror-γt showed a significant difference of both groups vs. control with *p* < 0.0017 for CD, and *p* < 0.0004 for UC. Indeed, it was significant the difference between UC and CD with *p* < 0.0081

To better understand the differences in T cell phenotyping and in cytokine production found in the CD and UC patients, flow cytometry was used to investigate the transcription factor expression of three genes involved in Th1, Th17, and Treg differentiation, corresponding respectively to T-bet, FoxP3, and Ror-γt. CD3^+^ T lymphocytes were gated forward side and the percentage of the selected transcription factor was analyzed (Fig. 3a). T-bet and Fox P3 showed a significant difference only for UC vs. control, with a *p* < 0.0272 and *p* < 0.0001, respectively. No significant difference was determined for the CD group. Ror-γt showed a significant difference for both groups with *p* < 0.0017 for CD, and *p* < 0.0004 for UC. Indeed, the difference between UC and CD was significant (*p* < 0.0081).

### NMR metabolomics

NMR metabolomics using both fingerprinting and targeting analysis [11, 23] of ^*1*^*H*, ^*1*^*H*_*T*_*2*_, and ^*1*^*H_Dfilter* spectra was applied to find potential biomarkers of disease for patients with Crohn’s disease (ILEUM, COLON, and ILEOCOLIC) or ulcerative colitis (UC) with respect to control (CTRL). In what follows, we present only the results of samples whose analysis satisfied the criterium of spectral quality based on PCA (i.e., outlier samples were discarded) and that eventually lead to favorable separation of the groups by MSA and univariant analysis. Other possible combinations of the groups of samples under study satisfied the PCA criterium but did not lead to favorable separation of the groups by MSA and therefore were no longer considered. In this regard, only ILEOCOLIC group (IC) from the three Crohn types showed significant differences vs. CTRL. Similarly, UC also showed significant differences vs. CTRL. A full NMR data table of this work is available in the database: www.ebi.ac.uk/metabolights/MTBLS1726.

### NMR of ILEOCOLIC vs. CTRL groups

**Fig. 4:**
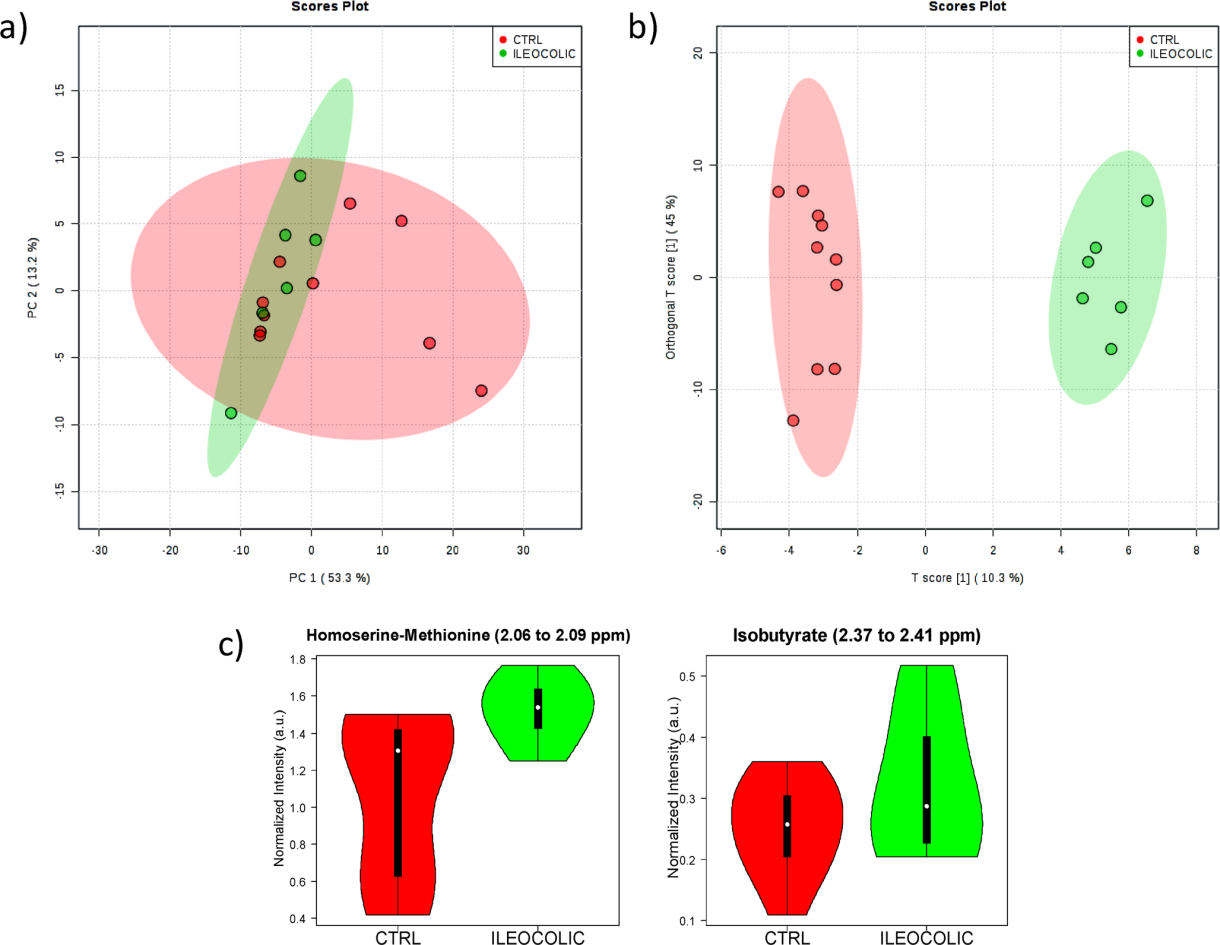
Statistics of the targeted analysis of the ^1^H_T_2_ spectra of plasma samples of patients with Crohn’s disease with ileocolic localization (ILEOCOLIC, *n* = 6) vs. control group (CTRL, *n* = 10). **a** PCA scores plot, **b** OPLS-DA scores plot (*R*^2^ 0.981 and *Q*^2^ 0.443) **c** Violin plot of the distribution of NMR intensities of the metabolites identified with differences between the two groups. Their bucket integrals in the spectra were found relevant for the OPLS-DA classification obtained in **b**

The best MSA classification of the ILEOCOLIC and CTRL groups was obtained with targeted analysis of the ^*1*^*H_T*_*2*_ spectra. The distribution of the PCA score plot for the two groups (see Fig. 4a) is highly compact and relatively random with no evident sample outliers that should be further considered. The OPLS-DA found two regions in the spectrum (buckets) with the maximum discrimination power corresponding to the metabolites homoserine/methionine and isobutyrate (Fig. S2a). The score plot of Fig. 4b shows excellent separation of the groups with no overlapping and the axes of the 2D scores cover 10.3 and 45% of the total variability.

The confusion matrix calculated from the cross-validated OPLS-DA model in Table 2 shows that 93.3% of unknown samples can be correctly classified by this method, a value that is substantially higher than the 50% expected by random. The ROC curve for homoserine/methionine is notably better for the classification than that for isobutyrate (Fig. S3), in agreement with their higher differences of intensity in the violin plots of Fig. 4c.

**Table 2:** Confusion matrix derived from the cross-validated OPLS-DA model, (a) for the classification of samples ILEOCOLIC vs. CTRL, (b) for the classification of samples UC vs. CTRL. One sample was randomly chosen for the cross-validation

From\to	CTRL	ILEOCOLIC	Total	% Correct
CTRL vs. ILEOCOLIC				
CTRL	9	0	9	100.00%
ILEOCOLIC	1	5	6	83.30%
Total	10	5	15	93.30%
CTRL vs. UC				
CTRL	6	3	9	66.70%
UC	2	5	7	71.40%
Total	8	8	16	68.70%

The univariant analysis of the NMR integral in the buckets corresponding to these metabolites Fig. 4c and Table 3 indicates a significant increment in the levels of homoserine–methionine with respect to CTRL (*p* < 0.04), and a more modest increment in the level of isobutyrate showing just a slight trend (*p* = 0.07).

**Table 3:** Relevant metabolites identified by NMR metabolomics in IC vs. CTRL and in UC vs. CTRL in serum samples. In IC, there is an increase of SCFA with respect to control as a consequence of anti-TNF treatment. In UC, the essential amino amide concentration is lower than in the control due to gut inflammation that leads to an intestinal malabsorption rate. Creatinine reduction in UC is IBD side effect associated with losing weight and muscular mass

Metabolite	HMDB ID	ILEOCOLIC	CTRL
ILEOCOLIC vs. CTRL			
Homoserie+methionine	HMDB00719, HMDB00718	High	Low
I sobutyrate	HMDB0001873	High	Low
UC vs. CTRL			
Proline	HMDB00162	Low	High
Creatine + creatinine	HMDB0000064, HMDB0000562	Low	High
Tryptophane	HMDB00929	Low	High

### NMR of UC vs. CTRL groups

**Fig. 5:**
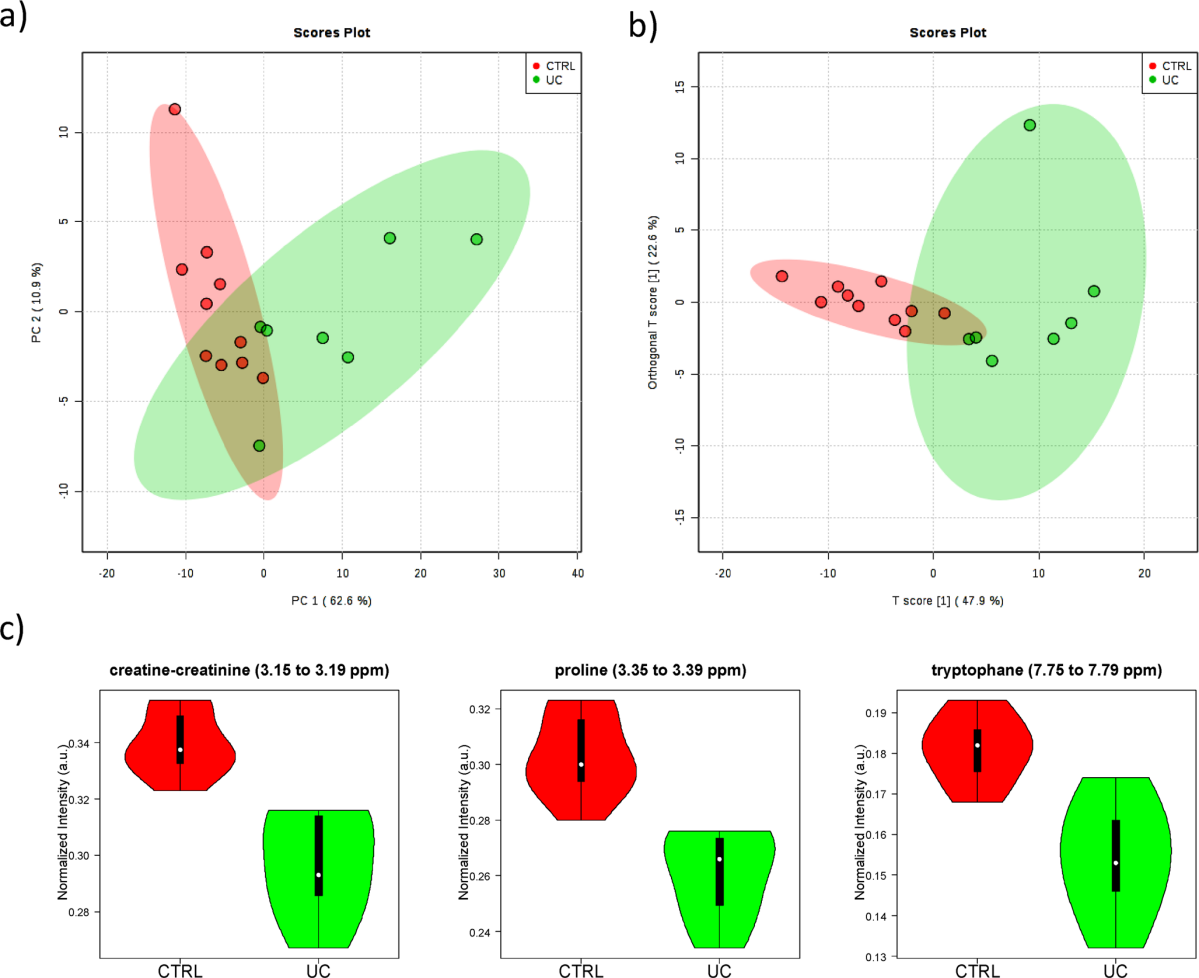
Statistics of the targeted analysis of the ^1^H spectra of plasma samples of patients with ulcerative colitis (UC, *n* = 7) vs. control group (CTRL, *n* = 10). **a** PCA scores plot, **b** OPLS-DA scores plot (*R*^2^ 0.796 and *Q*^2^ 0.315), **c** violin plot of the distribution of NMR intensities of three metabolites identified with differences between the two groups. Their bucket integrals in the spectra were found relevant for the OPLS-DA classification obtained in **b**

The best MSA classification of the UC and CTRL groups was obtained with targeted analysis of the ^1^H spectra. The distribution of the PCA score plot for the two groups (see Fig. 5a) is highly compact and relatively random with no evident sample outliers that need to be further considered.

The OPLS-DA found three regions in the spectrum (buckets) with the maximum discrimination power corresponding to the metabolites creatine/creatinine, proline, and tryptophane (Fig. S2b). The OPLS-DA score plot of Fig. 5b shows a good separation of the groups and the axes of the 2D scores cover 47.9 and 22.6% of the total variability. The confusion matrix calculated from the cross-validated OPLS-DA model in Table 2 shows that 68.7% of unknown samples can be correctly classified by this method, a value that is notably higher than the 50% expected by random. The ROC curve for the three aforementioned metabolites allows a remarkably good prediction of the groups (Fig. S4) in agreement with their marked differences of intensity in the violin plots of Fig. 5c.

Three metabolites were identified by analysis of the most relevant buckets of OPLS-DA: creatine-creatinine, proline, and tryptophan. The univariant analysis of the NMR integral in the buckets corresponding to these metabolites Fig. 5c and Table 3 indicates a significant decrease for UC patients in the levels of creatinine-creatinine (*p* < 0.0001), proline (*p* < 0.0001), and tryptophan (*p* < 0.0015), the last-mentioned being an essential amino acid absorbed from the diet. This group showed a higher inflammation rate that anti-TNF did not counteract.

## Discussion

IFX has been shown to be an effective treatment for patients with CD, but there is limited information about predictors of response to this medication. Identifying predictors of response to IFX under real clinical conditions would be of great benefit to the selection of patients for this treatment, and could in turn affect cost-related issues.

The use of NMR metabolomics of several types of samples including biofluids, intestinal biopsies, or feces for diagnosis and monitoring of IBD, has been recently reviewed [17]. In some cases, the comparison between remission and active patients resulted in a good PLS-DA modeling, with the identification of several discriminant metabolites such as N-acetylated compounds and amino acids such as phenylalanine [27].

This present study was designed to distinguish between CD and UC under anti-TNF treatment. Flow cytometry analysis of peripheral fresh blood samples was used to achieve a snapshot of the adaptive immune system cells that contributed to trigging inflammation. Furthermore, NMR metabolomics of blood serum was used to determine metabolites biomarkers for these diseases. The correlation of the data obtained by flow cytometry and NMR metabolomics permitted the stratification of both groups of patients.

The immuno-phenotyped results demonstrated that CD and UC patients had low blood concentration of Th1 subtypes (Fig. S1b). It was previously demonstrated that CCR5 is responsible of the T homing regulation process that allows CCR5 expressing T lymphocyte to migrate through the gut barrier both in IBD murine model and in human uveitis model [28, 29]. Therefore, a low percentage of CCR5 T cells in the blood could set off the decrease of the inflammation rate in the gut as the migration process would be prevented. However, normal Th1 values in the blood can be also a result of IFX treatment which involves the inactivation of Th1 proliferation by triggering apoptosis and inhibit NFκB pathways [30]. Thus, the treatment with IFX would benefit both CD and UC, as we observed normal percentages of Th1 (Fig. S1).

With regard to Th17 cells, these are crucial mediators of both CD and UC. Th17 infiltrates into the gut and produces high amounts of IL-17 and other cytokines, enhancing the inflammatory process [31]. In line with these findings, our results indicated that peripheral blood Th17 increased in both group of patients (Fig. 1c) with a higher percentage in UC. This could be explained because UC is considered a Th17 shifted disease [31]. Indeed, Th17 can rapidly convert into INF-γ producing Th1 or Treg cells due to their functional plasticity [31]. According to our findings, Treg significantly increased in UC (Fig. 1c), but no effect was observed regarding INF-γ (Fig. 2b), probably because Th1 was selectively inactivated by the treatment. It is clear from these results that Th17 and Treg cells appear more activated in the UC than CD patients in clinical remission.

Th cells are also characterized by a specific cytokines profile; thus, Th1 produces TNF-α and INF-γ, while Th17 leads to IL-17. We expected IL-17 to increase in both groups and with no significant effect on TNF-α; however, we observed an increase of TNF-α production in both CD and UC. This was probably due to the contribution of the CD8^+^ T cells, as T cells were gated in CD3^+^ that include CD4^+^ and CD8^+^ T cells (Fig. 2a). In line with these findings, Guo et al. demonstrated that IFX determines the inhibition of cytokine production, such as TNF-α and INF-γ through the downregulation of the NFκB signaling pathway in CD4^+^ T cells [30].

Transcription factor expression (Fig. 3b) showed a reduction of T-bet that could be a direct effect of IFX. It is known that IFX activates apoptosis in Th1 cells [30], thus reducing their total amount (Fig. S1). Moreover, transcription factor expression of Ror-γt and FoxP3 (Fig. 3b), responsible of Th17 and Treg differentiation, was significantly higher in UC patients only. It seems plausible that the treatment with IFX induces a different modulation in the immune response of IBD patients [32].

NMR metabolomics detected the increase in homoserine/methionine and isobutyrate levels for IC in serum. It is probably associated with the anti-TNF treatment of these patients, as it has been reported that IFX restores the microbiota composition in the gut of CD patients, as well as its functional capabilities [33]. Homeostasis helps to increase the absorption in the gut which is known to be positively correlated with methionine absorption and to the increase in bacterial secondary metabolites like butyrate [33]. It is also known that bacterial strains forming microbiota are able to extract from our diet a large variety of beneficial compounds, such as vitamins, essential amino acids, and short-chain fatty acids (SCFA) as products of their secondary metabolism. They could explain our NMR observation of increased levels of homoserine/methionine and isobutyrate in serum, due to the beneficial effects of IFX on microbiota and gut absorption [33–35]. Indeed, several studies demonstrated IFX anti-inflammatory effects, and its ability to heal gut lesions, downregulating apoptosis in intestinal epithelial cells (IEC), ultimately restoring tight intestinal junctions [33–36]. All these evidences could be involved in the improvement of the intestinal absorption and the increase of essential amino acids, as well as SCFA in CD patients.

The lower level of creatine detected by NMR in UC patients can be related with the health and physiology of skeletal muscle. It has been reported that this metabolite at the physiological level is associated with muscle hypertrophy and regulation of muscle repair upon damage. It carries out its effect by regulating mechanisms such as gene expression and inflammation in muscle [37]. Creatine is synthetized by an onset of three amino acids methionine, glycine, and arginine, and therefore, a decrease in the level of creatine can be accompanied by a reduction of essential amino acids, similar to that observed in our data for proline and tryptophan (Fig. 5c and Table 3). It has been reported that UC patients suffer a dysbiosis during intestinal absorption that ultimately may develop into sarcopenia, an IBD condition that implies skeletal muscle wasting [38]. It seems plausible that UC patients have a higher loss of gut integrity compared to CD, their gut barrier suffers from leaking, and thus, their ability to absorb and to retain nutrients from the diet is dramatically impaired which affects their normal metabolism and their health. Our NMR results show that UC patients suffer a dysbiosis in the absorption of essential amino acids and a reduction in the serum creatine level.

Indeed, our cytometry results of CD3^+^ T cell cytokine production shown in Fig. 2b indicates a high inflammation rate regarding IL-17 and TNF-α production for UC compared to CD. These data were corroborated by the transcription expression profiling of T-bet and Ror-γt (Fig. 3b) which also showed altered values, especially for UC patients. Moreover, high level of Treg (Fig. 1d) and transcription factor FoxP3 (Fig. 3b) (the transcription factor specifically expressed by these T cell subtype) can be associated with the inflammatory condition observed, as was previously demonstrated by Di Giovangiulio et al. (2019) [39], hence suggesting that T reg might contribute to inflammation rather than suppress it by activating T-bet transcription.

Although the enrolled patients were under clinical remission, the data reported here for CD and UC showed differences in the immune system cell activation as well as a metabolomic profile where high levels of homoserine-methionine and isobutyrate were associated with CD with ileocolic localization, while low levels of proline and tryptophane were associated with UC. Thus, the overall results obtained demonstrate that the proposed methodology was able to find relevant biomarker predictors to differentiate CD and UC patients under remission. These biomarkers are useful for IBD discrimination; however, it is important to bear in mind that they do not represent the response to the treatment with drugs such as IFX. The patients selected represent the real clinical conditions which is clearly a more difficult scenario than the ideal case of a clinical trial in which most of the variables are under control.

## Conclusions

The NMR metabolomics study of serum samples of CD and UC patients has identified several biomarkers that characterize IBD patients under remission. Significantly higher levels of homoserine-methionine and isobutyrate, with respect to control, were found to be characteristics of CD with ileocolic localization. For UC patients, creatine-creatinine, proline, and tryptophan levels were found significantly lower with respect to control.

The differences found by NMR correlated consistently with the immunological data, providing a robust pattern to distinguish CD and UC in remission patients with IFX treatment. The methodology proposed can potentially benefit several aspects of IBD treatment including diagnosis under real clinical conditions and may also aid to reduce the need for invasive tests such as colonoscopy.

Given the limited number of samples studied, further work needs to be done to understand the underlying mechanisms that promote total or partial remission of these diseases. Further information could possibly be obtained from the analysis of other biofluids (e.g., urine and plasma) and biopsies. They could possibly aid to find additional biomarkers or predictors for the diagnosis, to improve the therapeutic strategy and the follow-up response to the treatment. Future studies could complement these results with a validation of the biomarkers using a new cohort. Such improvement would make this methodology useful for the clinical practice to evaluate the response based on traditional criteria with molecular markers.

## Supplementary information

ESM 1 (DOCX 860 kb)

## Data Availability

Data base: www.ebi.ac.uk/metabolights/MTBLS1726
