# Supplementary material for: Targeted 1H NMR metabolomics and immunological phenotyping of human fresh blood and serum samples discriminate between healthy individuals and inflammatory bowel disease patients treated with anti-TNF
Source: J Mol Med (Berl). 2021 May 21;99(9):1251–64. doi: 10.1007/s00109-021-02094-y (PMC8367886; doi:10.1007/s00109-021-02094-y)
Supplement: Supplementary file 1 — (DOCX 860 kb) [file 109_2021_2094_MOESM1_ESM.docx]

SUPLEMENTARY DATA

**Targeted ^1^H NMR metabolomics and immunological phenotyping of human fresh blood and serum samples discriminate between healthy individuals and inflammatory bowel disease patients treated with anti-TNF**

Sara Notararigo^a^, Manuel Martín-Pastor^b^, Juan E. Viñuela Roldán^a^, Adriano Quiroga^a^, J. Enrique Dominguez-Munoz^a^, Manuel Barreiro-de Acosta^a^.

^a^ Instituto de Investigación Sanitaria de Santiago (IDIS), Complejo Hospitalario Universitario de Santiago (CHUS), Servicio Gallego de Salud (SERGAS), Santiago de Compostela, A Coruña 15706 (Spain)

^b^ Unidade de Resonancia Magnética, RIAIDT, CACTUS, Univ. Santiago de Compostela, A Coruña 15782 (Spain)

**Corresponding author**

Sara Notararigo: [sarita.not@hotmail.com](mailto:sarita.not@hotmail.com)

ORCID ID: 0000-0001-9841-5500

**Figure S1.** CD4^+^ T lymphocyte phenotyping. a) Representative cytometric pseudo-color and contour plot, from a CD patient. CD4^+^ T cell gated forward side were 55.2%. Total Th1 are 38.8%, 17.9% are CD4^+^CXCR3^+^, and 20.9% are CD4^+^CXCR3^+^ CCR5^+^ and b) Scatter dot plot, mean with SEM represented. No significant difference was found for both Th1 subtypes, indicating that treatment with IFX had a positive effect on Th1.

**Figure S2.** ^1^H_T_2_filtered spectrum of serum plasm of a control sample. The relevant spectral regions (buckets) found by OPLS-DA that better distinguish between the groups are marked in the spectrum together with the assignment of the metabolite/s. a) CTRL vs. ILEOCOLIC groups and b) CTRL vs. UC groups.

**Figure S3.** ROC curve (left) and plot of the range of normalized intensity (right) calculated from the normalized NMR signal area of a metabolite identified by the Targeted Analysis of ^1^H_T_2_ spectra of plasm samples of CTRL vs. ILEOCOLIC groups. a) Homoserine/methionine and b) isobutyrate. In all cases the criterium for true-positive is that the normalized area of the metabolite is higher for the ILEOCOLIC than the CTRL group.

**Figure S4.** ROC curve (left) and plot of the range of normalized intensity (right) calculated from the normalized NMR signal area of a metabolite identified by the Targeted Analysis of ^1^H spectra of plasm samples of CTRL vs. UC groups. a) Creatine/creatinine, b) proline and c) tryptophane. In all cases the criterium for true-positive is that the normalized area of the metabolite is lower for the UC than the CTRL group.
